# Cardiac metastasis from yolk sac tumor: case report and review

**DOI:** 10.1186/2162-3619-2-13

**Published:** 2013-04-30

**Authors:** Maria Carmo Pereira Nunes, Daniel Ribeiro Moreira, Teresa Cristina Abreu Ferrari

**Affiliations:** 1 Department of Internal Medicine, School of Medicine of the Federal University of Minas Gerais, Av. Professor Alfredo Balena, 190, Santa Efigênia, Belo Horizonte, MG, 30130 100, Brazil; 2 Department of Patology and Legal Medicine, School of Medicine of the Federal University of Minas Gerais, Belo Horizonte, MG, Brazil

**Keywords:** Cardiac metastasis, Germ cell tumors, Yolk sac tumors, Right ventricular mass

## Abstract

Cardiac metastasis of germ cell tumors is extremely rare, particularly in females. We report a case of a 26-year-old previously healthy woman who presented with a 5-month history of abdominal pain, weight loss, fever, generalized lymphadenopathy, and acanthosis nigricans. Biopsy of cervical lymph nodes revealed a poorly differentiated neoplasm. Immunohistochemical staining was positive for alpha-fetoprotein suggesting the diagnosis of a germ cell tumor. During the investigation, the patient developed heart failure and a mass attached to the right ventricle was detected by the echocardiogram. In a few days, she developed multiple organ failure and died. Post-mortem examination revealed a malignant mixed germ cell tumor of the right ovary with extensive hematogenic and lymphatic dissemination, a polypoid mass attached to the right ventricle, emboli in the endocardial and epicardial vessels, and infiltration surrounding the coronary arteries. To the best of our knowledge this is the third report of grossly visible heart metastases from a yolk sac tumor in a female patient. A summary of all published cases of germ cell tumors with cardiac metastasis over the last 20 years is also presented.

## Background

Ovarian germ cell neoplasm includes tumors with multiple histological patterns, and variable biologic behaviors [1]. These tumors grow rapidly, are usually unilateral and confined to one ovary in two thirds of the cases, and predominate in girls and young women. Mixed germ cell tumors consist of two or more admixed types of ovarian germ cell neoplasms and account for 5.3% of all malignant ovarian germ cell tumors [2]. Components of dysgerminoma mixed with endodermal sinus tumor (also called yolk sac tumors) are found most commonly. As a highly malignant neoplasm, yolk sac tumors metastasize at an early stage as well invade the surrounding structures and organs [1,3-6].

Although cardiac metastases can arise from different cancers, they are more frequently associated with carcinoma of the lung, breast and esophagus (due to the higher prevalence of these tumors), hematological malignancies including lymphoma and leukemia, and malignant melanoma, which shows the highest affinity to metastasize to the heart [4]. Cardiac metastasis of germ cell tumors is extremely rare, particularly in the female gender. We hereby report a case of a 26-year-old woman with mixed germ cell tumor of the right ovary who presented with widespread metastases including to the right ventricle. Post-mortem examination also showed neoplastic emboli in the endocardial and epicardial vessels and infiltration surrounding the coronary arteries.

## Case presentation

**Figure 1:**
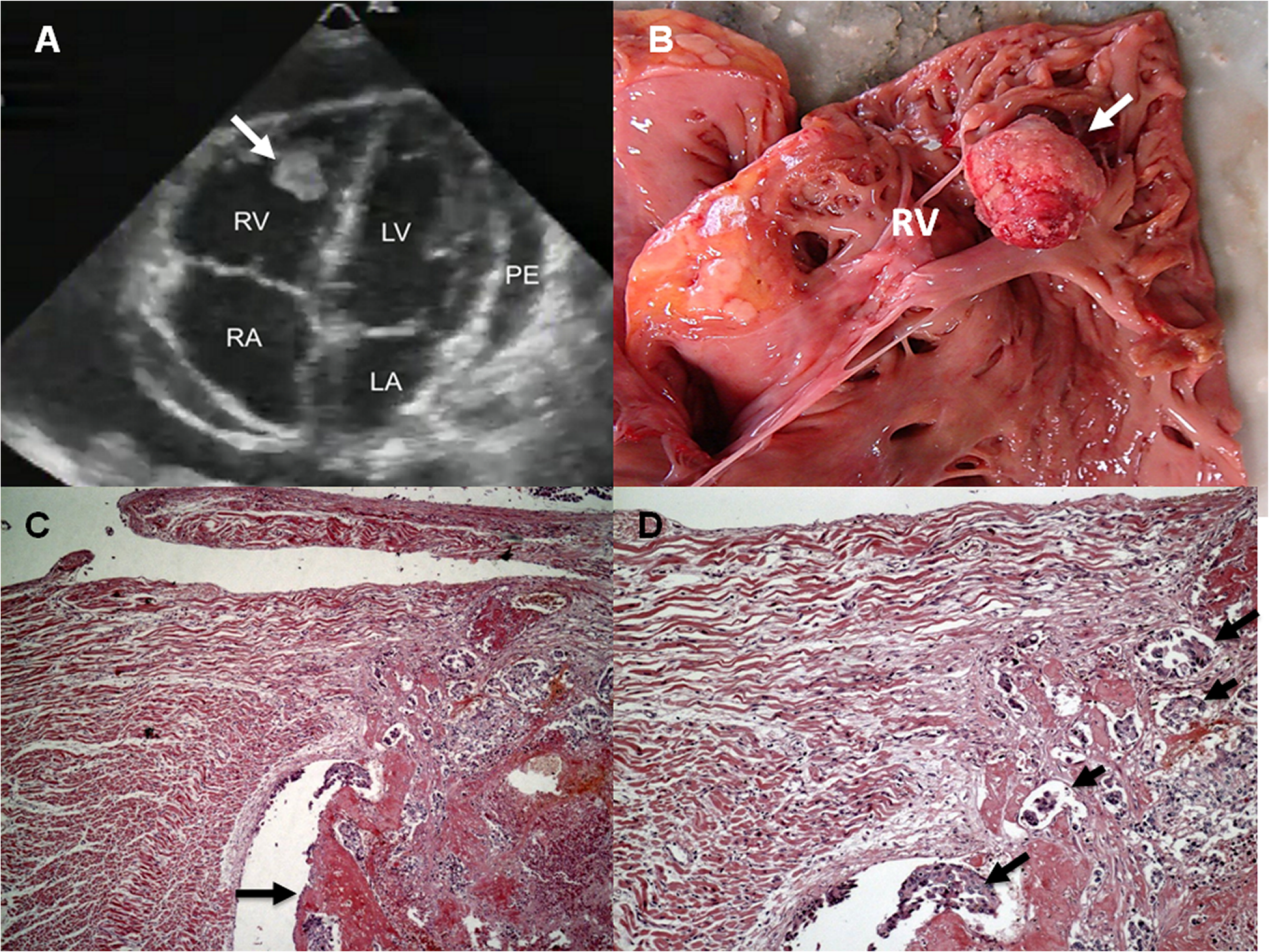
**Cardiac involvement by the tumor. A**: Image from transthoracic echocardiogram showing a mass attached to the apex of the right ventricle with a pericardial effusion (white arrow). RV = right ventricle; RA = right atrium; LV = left ventricle; LA = left atrium; PE = pericardial effusion. **B**: Photography derived from the autopsy showing opened right ventricle with a mass attached to the endocardial surface of the right ventricle (white arrow). RV = right ventricle. **C**: Polypoid cardiac mass (× 250) (black arrow). **D**: Emboli of neoplastic cells in myocardial and endocardial vessels (× 400) (black arrows).

A 26-year-old previously health woman presented with a 5-month history of abdominal pain, weight loss, fever, generalized lymphadenopathy, and acanthosis nigricans. Except for anemia (hemoglobin 11.1 g/dl), blood counts were within the normal ranges. Serological tests for human immunodeficiency virus (HIV), hepatitis viruses, syphilis, toxoplasmosis and cytomegalovirus were negative. Blood chemistry was unremarkable, and blood and urine cultures were negative. Thoracic, abdominal and pelvic computed tomography (CT) showed extensive lymphadenopathy and a solid mass with ill-defined borders near the uterus. The ovaries were not seen. Biopsy of cervical lymph nodes revealed a poorly differentiated neoplasm. Immunohistochemical staining was positive for alpha-fetoprotein suggesting the diagnosis of a germ cell tumor. During the investigation, the patient developed heart failure. A transthoracic echocardiography showed right ventricular (RV) dilatation, pericardial effusion, and a mobile mass attached to the endocardial surface of the apex of the RV (Figure 1, panel A). In the following days, she developed multiple organ failure and died before she could be subjected to any type of anticancer treatment. Post-mortem examination revealed a malignant mixed germ cell tumor of the right ovary with predominance of endodermal sinus (yolk sac) tumor elements, and extensive hematogenic and lymphatic dissemination to the lungs, kidneys, liver, intestines, uterine tube, mediastinum, and thyroid gland. A polypoid mass was found attached to the apex of the RV (Figure 1, panels B and C). Neoplastic emboli in the endocardial and epicardial vessels, and infiltration surrounding the coronary arteries were also detected (Figure 1, panel D).

The guardian of the patient provided written informed consent in accordance with the Declaration of Helsinki.

## Conclusions

Intracardiac metastasis is an extremely rare manifestation of germ cell tumors. It occurs by hematogenous spread, direct invasion from neighboring chest tumors or through the pericardial space, usually to the right side of the heart [3-6]. In our case, the neoplastic cells probably reached the heart hematogenously, through the inferior vena cava. Although there were mediastinal metastases, they did not present any continuity with the cardiac lesions.

In a review of the cases of germ cell tumors with cardiac metastasis published over the last 20 years (Table 1) [7-24] we found 17 cases: 16 of them were male with testicular seminoma or nonseminomatous germ cell tumor, and only one case of a yolk sac tumor was reported in a female child of 18 months old. In 11 of these reported cases, the metastasis was located in the right-side of the heart; in four, in a left-side chamber; and in two, in the pericardium. Five of the 17 patients died early in the chemotherapy treatment, and in eight, the disease was in remission at the time of the publication. Surgical resection of the metastasis followed by chemotherapy was the therapeutic approach performed in most cases.

**Table 1:** Review of documented cases of germ cell tumors with intracardiac involvement

**Reference (Author/year)**	**Gender/age (years)**	**Primary tumor (Histological type)**	**Time between diagnosis of primary tumor and cardiac metastasis**	**Cardiac location**	**Clinical manifestation of cardiac metastasis**	**Management/Outcome**
Savarese et al., 1995 [7]	Male/25	Testicular nonseminomatous mixed germ cell tumor	At diagnosis of the primary tumor	Right atrium extending to right ventricle	Severe low back and epigastric pain, painless swelling of the left, testicle, and a 20-pound weight loss	Surgical resection; 2 years without evidence of recurrence
Bath et al., 1997 [9]	Female/1.5	Yolk sac tumor	At diagnosis of the primary tumor	Intrapericardium infiltrating the right atrium	Lethargy, anorexia, tachypnea, cardiomegaly on chest x-ray and pericardial effusion on echocardiogram	Chemotherapy; remission 1 year following completion of treatment
Vohr et al., 1999 [10]	Male/21	Testicular nonseminomatous germ cell tumors	At diagnosis of the primary tumor	Right atrium	Syncope	Surgical resection; 1 year after cardiac surgery, a retroperitoneal metastasis was detected;
Low et al., 1999 [11]	Male/14	Testicular embryonal carcinoma with extensive vascular emboli	4 months	Left atrium	Breathlessness for several weeks before hospital admission and dilated veins over the upper thorax	Patient died 6 months after initial diagnosis
Deck et al., 2000 [12]	Male/20	Testicular nonseminomatous mixed germ cell tumor	3 years	Tricuspid valve	A new systolic ejection cardiac murmur	Tricuspid valve replacement; 1 year without evidence of recurrence
Singh et al., 2000 [13]	Male/27	Yolk sac tumor	At diagnosis of the primary tumor	Left atrium	Acute lower limb ischemia	Emergency excision of the tumor; patient died 5 weeks after initial presentation
Alaeddini et al., 2001 [14]	Male/26	Mixed germ cell testicular tumor (components of yolk sac, embryonal carcinoma, seminoma, and teratoma)	4 years	Right atrium that prolapsed through the tricuspid valve into the right ventricle	Right-sided heart failure	Surgical resection with tricuspid valve replacement.
Stefka J., 2003 [15]	Male/40	Testicular mixed nonseminomatous germ cell tumor (metastatic sarcomatoid germ cell tumor)	10 years	Right atrium protruding through the tricuspid valve up to the pulmonary valve	Shortness of breath	Surgical resection
Weinberg et al., 2004 [16]	Male/26	Testicular seminoma and smaller amounts of choriocarcinoma, teratoma, yolk sac, and embryonal carcinomas	At diagnosis of the primary tumor	Left ventricle	Respiratory distress and stroke	Chemotherapy; patient died 6 months after initial diagnosis
May et al., 2006 [17]	Male/42	Testicular germ cell tumor	At diagnosis of the primary tumor	Right atrium extending from the superior vena cava	Left-sided thoracic pain, shortness of breath on exertion, painless swelling of the left testicle, and a 20-pound weight loss	Surgical resection; stable for 12 months after surgery
Fujimura et al., [18]	Male/30	Testicular seminoma	12 years	Pericardium	Shortness of breath	Sudden death
Liu et al., 2007 [19]	Male/51	Testicular nonseminomatous germ cell tumor	At diagnosis of the primary tumor	Right atrium extending into the right ventricle and pulmonary arteries bilaterally	Progressive shortness of breath and pleuritic chest pain	Surgical resection; 17 months from initial presentation clinically free of disease.
Avasthi et al., 2008 [20]	Male/21	Testicular nonseminomatous mixed germ cell tumor	2 years	Left atrium	Shock	Died within an hour of admission
Taghavi et al., 2010 [21]	Male/32	Testicular nonseminomatous germ cell tumor	At diagnosis of the primary tumor	Right ventricle with an extension into the right atrium	Emergency department very short breath	Chemotherapy; CT-scan at 12-month follow-up revealed complete resolution of the cardiac lesion
Gursu et al., 2011 [22]	Male/17	Testicular nonseminomatous germ cell tumor	At diagnosis of the primary tumor	Right atrium	Right-sided heart failure	Surgical excision; stable for 6 months after surgery
Achouh et al., 2012 [23]	Male/32	Testicular mixed germ cell tumor	Around 1 year	Right atrium	Thrombosis of superior vena cava and right atrium mass	Not available
Jonjev et al., 2012 [24]	Male/24	Testicular mixed germ cell tumor (yolk sac malignant cells with large and pleomorphic nuclei scattered with islands of cartilage)	2 years	Right atrium	Acute right-sided heart failure	Surgical excision; stable for 6 months after surgery
**Our case/2012**	Female/26	Mixed germ cell tumor of the right ovary with predominance of endodermal sinus (yolk sac) tumor elements	Metastasis diagnosis before the identification of the primary tumor	Right ventricle	Abdominal pain, weight loss, fever, generalized lymphadenopathy, and acanthosis nigricans	Died before chemotherapy

A more extensive review, without limiting period of time, revealed just another case of germ cell tumor with cardiac metastasis in female. It was a primary chorion carcinoma of the right ovary with metastases to the left atrium in a 35-year-old woman, who presented with multiple metastases and died of respiratory failure, three months after the diagnosis [25].

A high level of suspicion is needed to establish the diagnosis of cardiac tumors. Frequently, the tumor is found incidentally during evaluation for a seemingly unrelated problem. Cardiac metastases often occur late in the course of a malignant disease and produce clinical symptoms in only about 10% of the affected patients [4-6]. Site, size, and tendency to cause embolism determine the clinical findings. In symptomatic patients, a mass is commonly detected by imaging methods, especially echocardiography. As the symptoms may mimic other cardiac conditions, the clinical challenge is to consider the possibility of a cardiac tumor; so that the appropriate diagnostic test can be conducted. Specific signs and symptoms are generally determined by the location of the tumor in the heart and not by the histological type [4-6]. Cardiac tamponade is one of the earliest and most frequent symptoms. Cardiac malignant disease may also cause arrhythmias and heart failure. The classic triad includes: symptoms resulting from intracardiac obstruction, signs of systemic embolization, and systemic symptoms [5,6].

Recommended treatment for patients with advanced malignant ovarian germ cell tumors and non-dysgerminoma histology is maximal cytoreduction followed by platinum-based combination chemotherapy [26,27]. Emergency surgical resection of intra-cardiac metastases has been recommended in the compromised patient and can be lifesaving [21]. New technical modalities (e.g., modern echocardiography, computed tomography, magnetic resonance imaging) provide noninvasive visualization of the intracardiac mass, which assists in designing the surgical approach not only to eliminate tumor tissue, but also to restore or maintain the hemodynamic status [14,24]. Most patients treated aggressively will be long-term survivors even if they have advanced disease. However, despite the sensitivity of ovarian germ cell tumors to platinum-based chemotherapy, tumor volume still remains one of the most important prognostic factors for outcome, as evident in the case presented, where outcome was poor due to highly disseminated metastatic disease.

To the best of our knowledge this is the third report of grossly visible heart metastases from a yolk sac tumor in a female patient.

## Consent

Written informed consent was obtained from the mother of the patient for publication of this case report and any accompanying images.

## Competing interests

The authors declare that they have no competing interests.

## Authors’ contributions

All authors have made important contributions to the manuscript and have approved this final version.
